# A new heat propagation velocity prevails over Brownian particle velocities in determining the thermal conductivities of nanofluids

**DOI:** 10.1186/1556-276X-6-361

**Published:** 2011-04-27

**Authors:** Kenneth D Kihm, Chan Hee Chon, Joon Sik Lee, Stephen US Choi

**Affiliations:** 1 Department of Mechanical, Aerospace, and Biomedical Engineering, University of Tennessee, Knoxville, Tennessee 37996, USA; 2 Department of Mechanical and Mechatronics Engineering, University of Waterloo, Waterloo, ON, N2L 3G1, Canada; 3 World Class University (WCU) Multiscale Mechanical Design Division, School of Mechanical and Aerospace Engineering, Seoul National University, Seoul 151-742, Korea; 4 Department of Mechanical and Industrial Engineering, University of Illinois at Chicago, Chicago, IL 60607, USA

## Abstract

An alternative insight is presented concerning heat propagation velocity scales in predicting the effective thermal conductivities of nanofluids. The widely applied Brownian particle velocities in published literature are often found too slow to describe the relatively higher nanofluid conductivities. In contrast, the present model proposes a faster heat transfer velocity at the same order as the speed of sound, rooted in a modified kinetic principle. In addition, this model accounts for both nanoparticle heat dissipation as well as coagulation effects. This novel model of effective thermal conductivities of nanofluids agrees well with an extended range of experimental data.

## Findings on nanofluid thermal conductivity

A nanofluid [1] is defined as a mixture of nanosized particles suspended in liquid as the base fluid. The nanofluid is perceived as an extended scope of earlier efforts to study the effective thermal conductivity of multiphase systems containing microscale particle-embedded solid materials [2-4] and a solid dispersion in liquid [5].

Since the first article on measurements of the enhanced thermal conductivity of nanofluids (suspension of Al_2_O_3 _and CuO nanoparticles in either water or ethylene glycol) using the transient hot-wire technique was published in 1999 [6], a number of successive measurement studies have supplemented the original findings and extended the parametric variations affecting the level of conductivity enhancement [7-23]. These experimental examinations have revealed the parametric importance of thermal conductivity enhancement, including the volume concentration of nanoparticles and their sizes, clustering or aggregation effect, pH effect, surfactant effect, and the base fluid temperature. As a systematic approach, Chon et al. [24] have constructed an experimentally extrapolated equation that predicts the nanofluid conductivity in terms of the related parameters.

Despite these advances, however, the published studies on theoretical predictions [25-30] of the thermal conductivity enhancement of nanofluids continue to be controversial and far from comprehensive. Table 1 shows the chronological presentation of published theories predicting conductivities, either for particle-embedded solid materials or for nanofluids. The first attempt at mathematical modeling dates back to 1873 by Maxwell [2], who presented effective thermal conductivity for a heterogeneous solid material, consisting of spherical solid particles of thermal conductivity *k*_p _embedded in a continuous solid phase with thermal conductivity *k*_BF_. The volume concentration *f *of the embedded spheres is taken to be sufficiently small, such that the spheres do not interact thermally and the effect of the particle size is assumed negligible. In 1962, Hamilton and Crosser [3] extended Maxwell's model and incorporated a modification for non-spherical particles by the empirical shape factor *n*.

**Table 1:** Historical development of nanofluidic thermal conductivity models

Author	Thermal conductivity enhancement, *k_eff_*/*k_BF_*
Maxwell [2]	
Hamilton and Crosser [3]	
Xuan et al. [25]	
Jang and Choi [26]	
Kumar et al. [27]	
Prasher et al. [28,29]	
Patel et al. [30]	
Present model	****

*f *denotes the volume concentration, *n *is the empirical shape factor (*n *= 3 for sphere), and *C_α_*, *C_β_*, *C_γ_*, *C_δ _*and *m *are empirical constants. Suggested constants [26,27,29] are 18 × 10^6 ^for *C_α_*, 2.9 to 3.0 for *C_β_*, 40000 for *C_γ_*, and 2.4 to 2.75 for *m*

A number of alternative models have been proposed with the use of the Brownian motion-induced micro-convection in a nanofluid. By adding the second term to the Maxwell model, Xuan et al. [25] proposed a model incorporating the Brownian motion of nanoparticles in 2003. A year later, Jang and Choi [26] introduced the Brownian-motion-driven convection model and attempted to describe the temperature-dependency of nanofluid thermal conductivity. They assumed the Nusselt number (*Nu*) to be the product of Reynolds number (*Re*) and Prandtl number (*Pr*), i.e., *Nu *= *Re*^2^*Pr*^2^, based on the postulation of Reynolds number of the order of unity. However, this assumption is invalid because it is incorrect to neglect the first two terms, i.e., lower degree terms of *Re·Pr*, in the expression for the Nusselt number that Acrivos and Taylor [31] have derived for heat transfer from a spherical particle at low values of the Reynolds number.

Kumar et al. [27] also attempted to incorporate the nanoparticle thermal conductivity based on the Brownian velocity. However, their model failed as Keblinski et al. [32] asserted that "the Brownian motion mean free path of a nanoparticle in fluid (by Kumar et al.) is on the order of 1 cm, which is unphysical."

In 2005, Prasher et al. [28] developed a model combining the Maxwell-Garnett model [33] incorporating both the Kapitza resistance effect of particles with the surrounding medium and the effect of the Brownian motion-induced convection. Later, they expanded their theoretical prediction for nanofluid thermal conductivity by adding aggregation conductivity contributions for the convection enhancements [29]. However, they assumed a less justifiable Brownian velocity of nanoparticles as  based on the kinetic theory of *gas*, which is valid just for fine particles suspended in a dilute gas (Boltzmann constant *k_b _*= 1.3807 × 10^-23^J/K, the base fluid temperature *T*, the nanoparticle densify *ρ*_p_, and its diameter *d_p_*)--but not quite valid for nanoparticles suspended in liquid. Quite possibly because of this conflict, their model fits only to a subset of experimental data, e.g., agrees fairly well with Al_2_O_3 _nanofluid data, but fails to fit to CuO nanofluid data.

The effect of the Brownian motion-induced microconvection remains controversial among different research groups. Eapen et al. [34] strongly argued that microconvection around randomly moving nanoparticles does not influence the thermal conductivity of the nanofluid. In 2007, Das group proposed a nanofluid thermal conductivity model based on a cell model [30]. Their cell model tried to explain the nonlinear dependence of thermal conductivity of nanofluids on particle volume fraction. However, their empirical constants were defined only to fit to their experimental data. In fact, their model constants did not show consistency for an identical Al_2_O_3 _nanofluid.

The kinetic principle well describes the thermal conductivity of gas, as the gas molecules are assumed to be freely moving due to their relatively lean distributions [35]. For liquids, however, their stronger intermolecular forces, primarily because of the higher packing density, make it necessary to modify the kinetic theory. In addition, the molecular collision velocities of gases are too low to explain liquid thermal conductivities that are at least one order of magnitude higher than the gas conductivities. Hence, the thermal conductivities of denser liquids are conjectured to be more properly expressed by the faster sound propagation in the case of liquids, and by the phonon velocity in the case of solids.

In this article, a novel theoretical model describing the nanofluid thermal conductivities, considering all major effective parameters including the size, density and volume concentration of nanoparticles, the fluid temperature and viscosity, and relevant thermal parameters such as thermal conductivity of base fluid and heat capacity of nanoparticles, is proposed and examined for its validity against available experimental data.

## Introduction of heat propagation velocity

The enhanced thermal conductivity of a liquid suspension containing highly conductive metal or metal-oxide particles, such as nanofluids with Au, Al_2_O_3_, or CuO, is believed to be attributed to the interaction of nanoparticles with the base fluid molecules. The thermal conductivity of a liquid is given by [36]:(1)

where ρ and *c*_v _are the liquid density and specific heat, respectively, *u *is sonic velocity in liquid, and *a *is the molecular travel distance between two successive collisions. Likewise, the thermal conductivity enhancement of a nanofluid can include the thermal properties of nanoparticles (*ρ*_p_, *c*_p_), the heat propagation velocity *V*_ht_, which substitutes the sonic velocity, the heat travel distance *l*_ht_, which replaces the collision travel distance *a*, and additional consideration of the volume fraction of nanoparticles *f *[14,35]:(2)

Note that the combined term *V*_ht_·*l*_ht _relates to the increase of thermal diffusivity of nanofluid as .

The heat travel distance *l*_ht_, which is defined as the freely traveled distance of heat energy during the interaction of base fluid molecules and nanoparticles, is shown to be equivalent to the root-mean-square displacement of nanoparticles [25] as:(3)

where *μ *is the dynamic viscosity of the base fluid and *c*_1 _is a dimensionless proportional constant. In the case of nanofluid, if *l*_ht _is assumed to have the same order of magnitude as the mean free path of water molecules, one can estimate *l*_ht _~ 0.170 nm.

The heat propagation velocity can be estimated by examining the order-of-magnitudes of the involved parameters in Equation 2. For example, for 47-nm Al_2_O_3 _at 1 vol.% concentration (*f*·*ρ*_p_·*c*_p _~ 3.2 × 10^4^), the thermal conductivity enhancement Δ*k*_enh _is found to range from 0.025 to 0.100 W/K m [24]. Thus, the heat propagation velocity *V*_ht _is estimated to be on the order of 10^3 ^m/s. While a more rigorous analysis to determine the heat transfer velocity is yet to be discussed, this estimation is consistent with the conjectures of the characterisitc heat propagation velocity being on the scale of the sound propagation velocity of an order 10^3 ^m/s of both in a liquid medium [22] and in a colloidal medium [37,38].

The heat propagation velocity *V*_ht _represents the heat propagation rate by the vibration of base fluid molecules. In a stationary liquid, individual molecules are constantly moving, and their motions are largely confined within a "cage" formed by the closely packed neighboring molecules [36]. This virtual cage is conceived by the energy barrier of height  where  represents the molar free energy of activation for escaping the cage and  denotes the molar Avogadro number. The molecular vibrational frequency *ν *is given by:(4)

where *k*_b _denotes the Boltzmann constant, *h *and *R *are the Planck constant and the specific gas constant, respectively, and *T *is the fluid temperature. The free energy of activation, , is assumed to be constant for a specified fluid and also assumed to be directly related to the internal energy of vaporization at the normal boiling point [39].

The internal energy is given from Trouton's rule [40] as  and  where  is the enthalpy of vaporization at the normal boiling point *T*_b_. Substituting this into Equation 4, and then multiplied by the the heat propagation length scale λ*_ht_*, gives an expression for the heat propagation velocity *V_ht _*as:(5)

The propagation length scale λ*_ht_*, is calculated based on the assumption that the base fluid moledules and nanoparticles are arranged in a cubic lattice, with a center-to-center spacing given by , where is molar mass of the base fluid.

## New model for nanofluid thermal conductivity

Substituting Equations 3 and 5 into Equation 2 gives an expression for the effective nanofluidic thermal conductivity *k*_eff _as:(6)

Two additional modifications of Equation 6 are implemented. First, the volume fraction *f *is modified to a reduced volume fraction *f^a ^*(*a *< 1) to account for the coagulation of nanoparticles that effectively reduce the original volume fraction [38]. The coagulation becomes more severe to require a smaller exponent *a *with increasing particle concentration because of the decreased inter-particle distance. For example, The surface-to-surface distance of nanoparticles is twice the particle size at 1 vol.%; however, it can decrease to half the particle size at 5 vol.%. Secondly, the effective thermal conductivity of Equation 6 is modified by multiplying the heat capacity ratio of the base fluid to nanoparticles, . It is known that shorter heat dissipation time from nanoparticles into the base fluid enhances the effective thermal conductivity of nanofluid [41,42]. The heat dissipation time decreases with increasing heat capacity of the base fluid and decreasing heat capacity of the nanoparticles. In other words, nanoparticles with a smaller heat capacity require shorter heat dissipation time to the base fluid, and this results in greater thermal diffusion and higher effective thermal conductivity. The effective conductivity increases in consistency with the heat capacity ratio .

Therefore, after accommodating the above two modifications, the effective thermal conductivity of nanofluids of Equation 6 is given by:(7)

where *C *is a modified constant and *c*_BF _is the base fluid specific heat. The heat transfer length scale *λ*_ht _is difficult to be calculated directly, but may be determined by order analysis and merged into the constant *C*. The exponents *a *and *b *are empirical constants that represent the effect of nanoparticle coagulation and of nanoparticle heat dissipation, respectively. A regression analysis of published experimental data by the authors [24] provides *a *= 0.70, *b *= 1.5, and *C *= 3.58 × 10^-14 ^m for the case of Al_2_O_3 _nanoparticles of three different sizes (11 nm, 47 nm, 150 nm diameters) suspended in water under various experimental conditions of a volume concentration range of 1 to 4 vol.% and a tested temperature range of 21 to 71°C.

**Figure 1:**
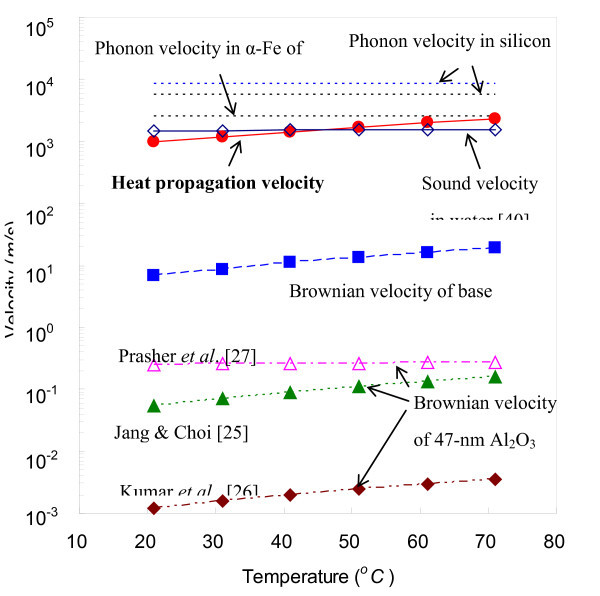
**Temperature dependence of Brownian velocities **[26-28], **speed of sound **[43], **phonon velocities **[44,45], **and the heat propagation velocity of the present model (Equation 5)**.

Figure 1 compares different types of velocity scales that are considered relevant in describing nanofluid thermal conductivity models: (1) three differently defined Brownian velocities for 47-nm Al_2_O_3 _nanoparticles [26-28], (2) the Brownian velocity of the base fluid (water) molecules [26], (3) the heat propagation velocity based on the currently proposed model (Equation 5), (4) the sound velocity in water [43], and (5) phonon velocities for selected solid mediums of αalpha-Fe and silicons [44,45]. The phonon velocities are expected to be faster than the heat propagation velocity in liquid because of the relatively higher heat conductivities in solid mediums.

Table 2 shows the functional expressions of these velocities and their calculated magnitudes for the tested temperature range of 21 to 71°C. Note that all the previously reported nanofluid thermal conductivity models use the Brownian velocities for the heat propagation velocity, while the present propagation velocity is comparable to the sonic velocity in the base fluid that is several orders larger than the Brownian velocities. The Brownian velocities based on the nanoparticles are too slow to be compatible with the relatively faster heat conduction phenomena in liquids. Furthermore, the Brownian velocities of the base fluid of water molecules are also considered too slow to properly model the nanofluidic conductivities.

**Table 2:** Differently defined Brownian velocities and heat propagation velocities, and their magnitudes calculated for the range from 20 to 71°C

Author	Velocity model	Calculated velocity (m/s)
Brownian velocity of nanoparticles [26]		0.055-0.160
Brownian velocity of nanoparticles [27]		0.0012-0.0035
Brownian velocity of nanoparticles [26]		0.249-0.270
Brownian velocity of water molecules [26]		6.710-19.534
Sound propagation velocity in water [45]		1480-1555
Heat propagation velocity [Present model, Equation 5]		950-2250

Nevertheless, we do not mean that the Brownian motion is not related to the thermal conductivity enhancement. Nor do we mean that Brownian convection is not significant. What we imply is that the assumption in [26], i.e., the Nusselt number can be expressed as *Nu *= *Re*^2^*Pr*^2^, is invalid because it is incorrect to neglect the first two terms, i.e., lower degree terms of *Re·Pr*, in the expression for the Nusselt number that Acrivos and Taylor [31] have derived.

In addition, in order to have significant convection effect by wavelength mode of long molecular motion, the bulk fluid needs externally imposed gradients such as pressure, gravity or temperature. However, a nanofluid has quiescent condition, which cannot support any convection [34,46]. The Brownian velocity, as shown in Figure 1, is several orders of magnitude lower than the required velocity scale of 10^3 ^in modeling nanofluid conductivity enhancement.

**Figure 2:**
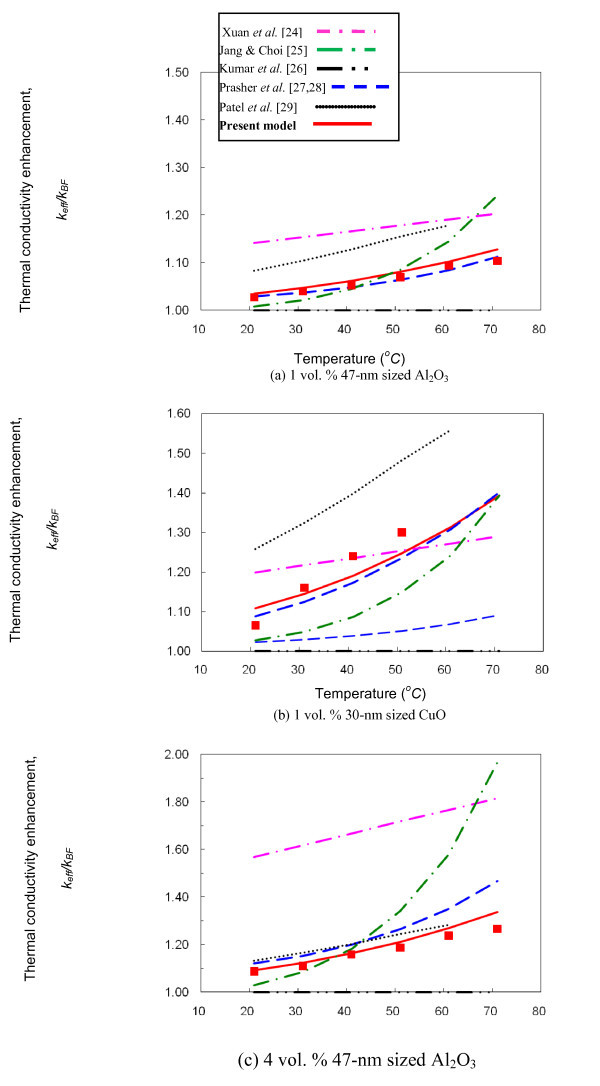
**Comparison of the present model (the solid curves) with published models **[25-29]**for the thermal conductivities of nanofluids**. The symbols represent the presently (CuO nanofluids) and previously (Al2O3 nanofluids [23]) measured conductivities from the University of Tennessee laboratory: (a) 1 vol. % Al2O3 nanofluid [13], (b) 1 vol. % CuO nanofluid [present experiment], and (3) 4 vol. % Al2O3 nanofluid [13].

Figure 2a-c shows the present model for thermal conductivities of water-based nanofluids, Equation 7, in comparison with five published models [25-30], for the three different nanofluids. The symbols represent the corresponding experimental data for Al_2_O_3 _[24] and CuO [present work]. For all three nanofluids with 47-nm Al_2_O_3 _at 1 and 4%, and 30-nm CuO at 1%, Xuan et al. [25] overestimated the Maxwell's model [2] for nanofluids. Jang and Choi's model [26] shows proximity with experimental data for up to about 50°C for 1 vol.% Al_2_O_3 _and 40°C for 4 vol.% Al_2_O_3_, but substantially deviates thereafter. This deviation beyond a certain temperature is believed to be attributed to their incorrect postulation implied in determining the Nusselt number, as previously noted. For the CuO nanofluid, their model shows large discrepancies throughout the tested temperature range. Additionally, the model by Kumar et al. [27] wrongly postulates the mean free path of the base fluid, as pointed out by Keblinski et al. [32], and completely fails to predict nanofuidic thermal conductivities for all presently tested conditions.

Prasher et al. [28,29] show fairly good agreement with the experiments for the Al_2_O_3 _nanofluid, as shown in Figure 2a, c. However, for the CuO nanofluid (Figure 2b), their model underestimates the corresponding experimental data [24]. When completely different model parameters were imposed for CuO from that of Al_2_O_3_, the model agrees well with the data; however, the model then lacks comprehensiveness because different model parameters need to be determined for different types of nanofluids. Finally, Patel et al. [30] agrees fairly well with the experimental data at higher concentrations (Figure 2c) but overestimate the thermal conductivities for low volume concentrations (Figure 2a, b).

**Figure 3:**
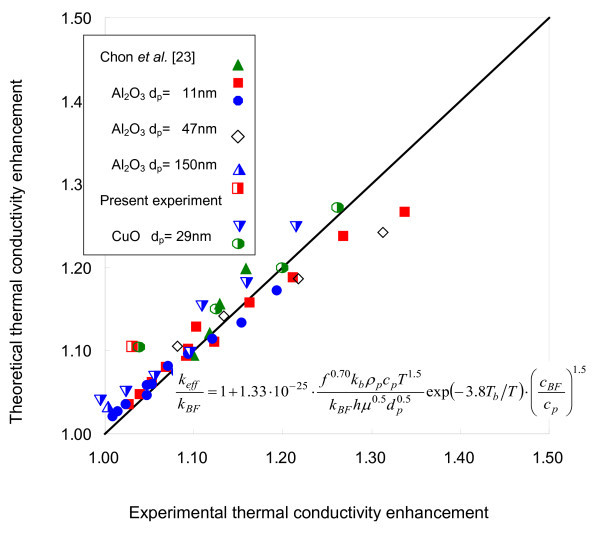
**Predictions of the present model with corresponding experimental data for various Al_2_O_3 _and CuO nanofluid samples **[6,10,24].

In contrast, the present model of Equation 7 shows consistent agreement with the experimental data not only for both nanofluids but for all the tested conditions of temperatures and volume concentrations. Furthermore, Figure 3 demonstrates the comprehensiveness of the present model of Equation 7 in comparison with published experimental data for both Al_2_O_3 _and CuO nanofluids from different leading groups [6,10,24].

## Concluding remarks

In order to alleviate the controversy associated with the relatively slow Brownian velocity of nanoparticles to describe the microconvection effect on thermal conductivities of nanofluids, a new and faster heat transfer velocity is proposed, on the same order as the speed of sound and rooted from a modified kinetic principle. Furthermore, the new model for effective thermal conductivities of nanofluids, which is based on the faster heat propagation velocity and accounts for both nanoparticle heat dissipation and coagulation as follows,(7a)

can more accurately and comprehensively describe the effective thermal conductivities of nanofluids with different types (Al_2_O_3 _and CuO nanofluids) and sizes of nanoparticles (ranging from 10 to 150 nm), for a relatively wider range of temperatures in comparison with the most popular range of up to 50°C of published studies.

As similar conceptual studies, the recent thermal-wave [47] and the dual-phase lagging heat conductions [48] are attracted by researchers because both models can explain the high-rate heat flux in microscale and also can be applied to the thermal conductivity of nanofluid. Thermal-wave and dual-phase lagging heat conduction are developed analytically, however the new model is approached by physical manner and it considers more practical factors such as particle coagulation effect and heat dissipation effect. Therefore our new model will be bridging the practical thermal conductivity enhancement of nanofluid and theoretical concept of the high-rate heat flux of nanofluid such as thermal-wave dual-phase lagging heat conduction of nanofluid.

## Competing interests

The authors declare that they have no competing interests.

## Authors' contributions

KK carried out the whole research project as PI/academic advisor of CC and drafted the manuscript. CC performed both theoretical studies and experimental measurements, and also participated in drafting the manuscript. JL participated in the WCU research project and design to complete the draft together with KK. SC conceived of the study, and participated in its coordination. All authors read and approved the final manuscript.
